# Influence of chemical ordering on the thermal conductivity and electronic relaxation in FePt thin films in heat assisted magnetic recording applications

**DOI:** 10.1038/srep32077

**Published:** 2016-08-26

**Authors:** Ashutosh Giri, Sung Hun Wee, Shikha Jain, Olav Hellwig, Patrick E. Hopkins

**Affiliations:** 1 Department of Mechanical and Aerospace Engineering, University of Virginia, Charlottesville, Virginia 22904, USA; 2 San Jose Research Center, HGST, a Western Digital company, San Jose, California 95135, USA; 3 Institute of Physics, Chemnitz University of Technology, D-09107 Chemnitz, Germany; 4 Institute of Ion Beam Physics and Materials Research, Helmholtz-Zentrum Dresden–Rossendorf, 01328 Dresden, Germany

## Abstract

We report on the out-of-plane thermal conductivities of tetragonal L1_0_ FePt (001) easy-axis and cubic A1 FePt thin films via time-domain thermoreflectance over a temperature range from 133 K to 500 K. The out-of-plane thermal conductivity of the chemically ordered L1_0_ phase with alternating Fe and Pt layers is ~23% greater than the thermal conductivity of the disordered A1 phase at room temperature and below. However, as temperature is increased above room temperature, the thermal conductivities of the two phases begin to converge. Molecular dynamics simulations on model FePt structures support our experimental findings and help shed more light into the relative vibrational thermal transport properties of the L1_0_ and A1 phases. Furthermore, unlike the varying temperature trends in the thermal conductivities of the two phases, the electronic scattering rates in the out-of-plane direction of the two phases are similar for the temperature range studied in this work.

The push towards high areal densities beyond 1 Tb/in^2^ in magnetic recording media has resulted in a pursuit for materials with higher magneto-crystalline anisotropy and higher thermal stability^1^,^2^,^3^. One such material that is sought after in the development of next generation hard disk drives for heat assisted magnetic recording (HAMR) is chemically ordered L1_0_ iron platinum alloy (FePt)^4^,^5^. In HAMR, along with a magnetic write field, local heating beyond the Curie temperature of the material is applied in order to lower the coercivity of the recording media during the write process. Therefore, accurate knowledge of the thermal properties and resulting temperature gradients of the FePt media are paramount in advancing HAMR technology.

Along with these considerations, the effect of varying chemical order from the cubic A1 phase to the tetragonal L1_0_ phase (as a result of high temperature deposition) in FePt alloys on their thermal conductivity still remains to be fully understood. Recently, Duda *et al*.^6^ have investigated the phononic thermal transport properties of Lennard-Jones (LJ) AB alloys with tetragonal L1_0_ and cubic A1 phases. By conducting nonequilibrium molecular dynamics (NEMD) simulations, they demonstrated that due to major differences in the crystallographic reconfiguration such as periodicity, atomic basis and symmetry between the tetragonal and cubic phases, the dominant scattering mechanisms can vary significantly between the different phases of the alloys, which can lead to very different thermal conductivities for the two phases^6^. So far, their computational findings and hypothesis have not been demonstrated experimentally.

In this work, we study the thermal properties of (001) textured, chemically ordered L1_0_ and disordered A1 phases of FePt thin films via the time domain thermoreflectance (TDTR) technique. The measured thermal conductivity at room temperature for the A1 phase is comparatively lower than the thermal conductivity of the L1_0_ phase along the [001] easy-axis direction, suggesting that the chemical ordering into alternating Fe and Pt planes in the out-of-plane direction can lead to as much as a 23% increase in the thermal conductivity of these alloys. NEMD simulations on model FePt structures support the experimentally observed thermal conductivity difference at lower temperatures between the A1 and L1_0_ phases, suggesting that the change in the thermal properties between the phases can be partially attributed to a difference in vibrational thermal conductivity; our NEMD simulations are conducted using simplified potentials for easy generalization. In the isotropic A1 phase, the electronic contribution to thermal conductivity most likely contributes to the majority of thermal conduction based on a comparison of in-plane electrical resistivity to TDTR measured out-of-plane thermal conductivity (albeit, the isotropy of this disordered alloy lends credence into our observation). However, this luxury of isotropy is not available in the L1_0_ structure, preventing the comparison of the in-plane resistivity to our measured thermal conductivity in the out-of-plane direction. We also examine the electron relaxation rates between the L1_0_ (001) and A1 phases in the out-of-plane direction by considering the early pump-probe delay times (immediately after laser pulse absorption on the metal surface); the two phases demonstrate similar electronic scattering rates in the out-of-plane direction even though the electrical conductivity measurements between the phases are vastly different in the in-plane direction.

## Results and Discussions

**Figure 1:**
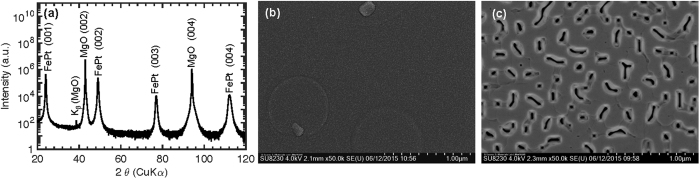
(**a**) Out-of-plane X-ray diffraction (XRD) patterns for the FePt alloy deposited at 1023 K. The pattern shows strong FePt (00*l*) peaks with high FePt (001)/(002) peak integral intensity ratio indicative of good chemical ordering. Plan-view SEM images of 100 nm thick FePt films on (100) MgO single crystal substrates deposited at (**b**) RT and (**c**) 1,023 K.

FePt films with ~100 nm thicknesses were grown on (001) oriented MgO single crystal substrates by using an AJA sputtering system. The degrees of chemical ordering of the films were controlled by deposition at different substrate temperatures: i.e., room temperature (RT) for the A1 phase and 1,023 K for the L1_0_ phase. Figure 1(a) shows the X-ray diffraction (XRD) pattern for the film with strong L1_0_ chemical ordering and (001) texture. This result confirms that the film has strong FePt (00*l*) peaks, a narrow FePt (002) rocking curve width (Δ*ω*) of ~0.56°, a high *I*_(001)_/*I*_(002)_ ratio of ~1.4, and a low *c*/*a* ratio of ~0.959. The film was also measured to have a curie temperature (*T*_*c*_) of ~783 K, comparable to reported *T*_*c*_ of ~750–770 K of the fully L1_0_ ordered FePt phase. Compared to the film deposited at 1,023 K, the film deposited at RT has a *T*_*c*_ of ~660 K close to that of A1 FePt phase (~650 K), suggesting that the film has disordered A1 FePt phase even though no FePt peaks are observable by a typical symmetric, out-of-plane *θ*-2*θ* XRD scan due to poor crystallinity and texture quality.

Scanning electron microscope (SEM) images show that the film deposited at RT is comprised of a 100% continuous FePt layer (Fig. 1(b)), whereas, the film deposited at 1,023 K has a well-connected structure with some surface pores that are caused by incomplete coalescence during the film growth (Fig. 1(c)). The dominant growth mechanism for FePt films grown on MgO substrates is 3D island (Volmer-Weber) growth mode considering their surface energy (*γ*_FePT_ ~ 2 J m^−2^, *γ*_MgO_ ~ 1.1–1.2 J m^−2^) and large lattice mismatch (~9.6%). In this growth mode, the film becomes continuous only at or above a critical thickness that is influenced by the surface energy and lattice mismatch differences as well as the substrate temperature during deposition^7^. Although a 50–100 nm thickness range has been reported to be a critical thickness for FePt films on (100) MgO deposited at ~973 K^8^, our results show that the thickness of ~100 nm is not sufficient to form a 100% continuous layer with no surface pores for FePt deposition on MgO at 1,023 K.

## Thermal Conductivity

**Figure 2:**
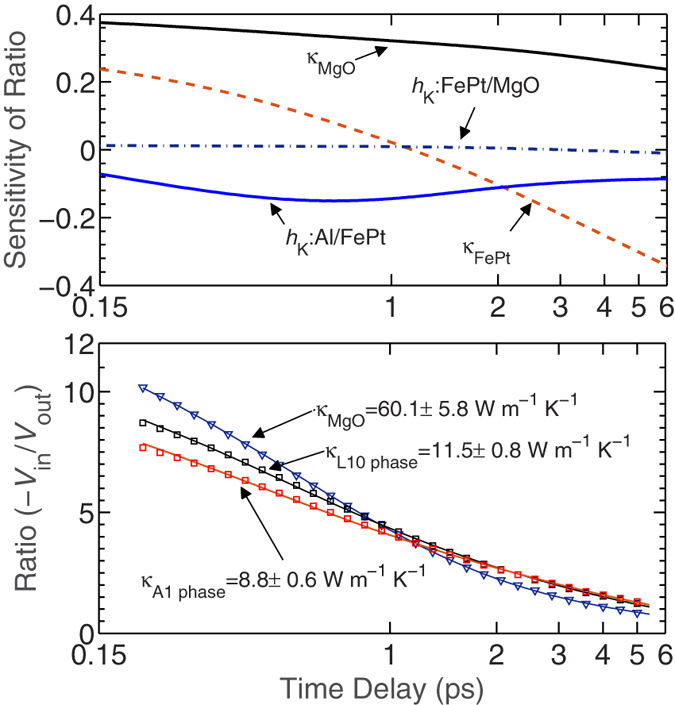
(**a**) Sensitivities of ratio (−*V*_in_/*V*_out_) to the out-of-plane thermo-physical properties of the L1_0_ phase FePt sample as a function of pump-probe time delay at 8.4 MHz pump modulation frequency. (**b**) Ratios (−*V*_in_/*V*_out_) and best-fit curves for the Al/MgO (blue triangles), Al/L1_0_ phase FePt/MgO (black squares) and aluminum/A1 phase FePt/MgO (red diamonds).

Figure 2 shows the sensitivity of the ratio of −*V*_in_/*V*_out_ to the various input parameters in the three-layer thermal model used to analyze the TDTR data^9^. For the pump-probe delay times from 150 ps to 6 ns, the thermal model is mostly sensitive to the thermal conductivities of the FePt thin film and the MgO substrate. Therefore, we make separate measurements on a control sample (Al/MgO) to determine the thermal conductivity of MgO and minimize the uncertainty in the determination of thermal conductivity for the FePt thin films. To this end, the thermal conductivity is determined through a two-layer model fit to the experimental data with the thermal boundary conductance across Al/MgO, *h*_k,Al/MgO_, and the thermal conductivity of MgO, *κ*_MgO_, as free parameters in the model. We measure the thermal conductivity of MgO to be 60.1 + 5.8 W m^−1^ K^−1^ as shown in Fig. 2b, which includes the experimental data as well as the best fit to the data following a least-squares fitting routine. The thermal boundary conductance across Al/MgO is measured as 178 ± 18 MW m^−2^ K^−1^. Figure 2b also shows the ratio of the TDTR signals for the Al/FePt/MgO samples along with the three-layer model fits.

We determine the thermal conductivities of the L1_0_ phase and the A1 phase to be 11.5 ± 0.8 W m^−1^ K^−1^ and 8.8 ± 0.6 W m^−1^ K^−1^, respectively. Our measurement of *κ* = 8.8 ± 0.6 W m^−1^ K^−1^ for the A1 phase at room temperature is consistent with the measurement of an A1 disordered FePt thin film (*κ* = 8.5 W m^−1^ K^−1^) in ref. 10, where they have used the frequency domain thermoreflectance technique to measure the thermal conductivity. However, our measurement of *κ* = 11.5 ± 0.8 W m^−1^ K^−1^ for the L1_0_ phase is slightly lower than that reported in ref. 11 (13 W m^−1^ K^−1^). This discrepancy could be due to the fact that the authors in ref. 11 did not use a transducer while performing their TDTR experiments, which can lead to significant error while analyzing the TDTR data to determine the cross plane thermal conductivities of thin films. Furthermore, the analytical procedure implemented in ref. 11 does not account for pulse accumulation effects and radial heat conduction in the film as well as in the substrate that can lead to significant errors if ignored in the analytical procedure as detailed in refs. 12, 13, 14.

**Figure 3:**
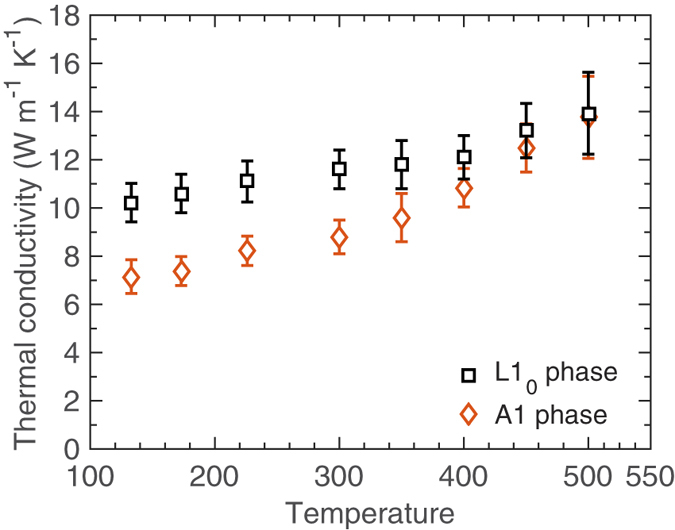
Out-of-plane thermal conductivities as a function of sample temperature for the L1_0_ ordered (black squares) and A1 disordered (red diamonds) FePt films.

Figure 3 summarizes our measurements for the samples over a range of temperatures (133 K–500 K). It is interesting to note that as the sample temperature increases beyond room temperature, the thermal conductivities of the L1_0_ and the A1 phases converge. This finding is consistent with the results obtained for LJ-based AB alloys reported by Duda *et al*.^6^, where it was shown that at relatively high sample temperatures, the thermal conductivity of the A1 disordered phase can be comparable to and even higher than that of the L1_0_ ordered phase. Note, by repeating our measurements at room temperature following the measurements at higher temperatures, we have confirmed that the thermal conductivities of the FePt samples are reversible, suggesting that the elevated sample temperatures during TDTR measurements do not alter the FePt alloy microstructures.

**Figure 4:**
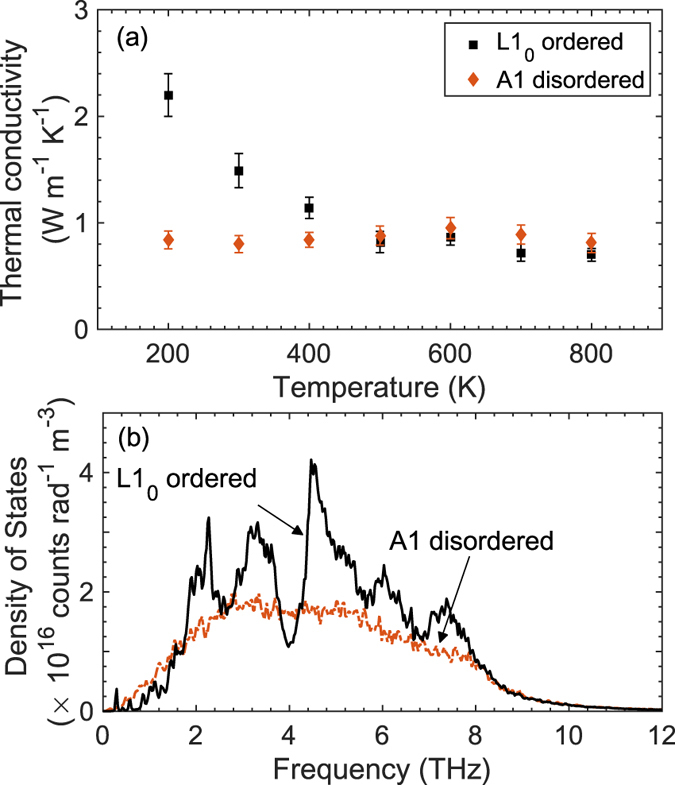
(**a**) MD-simulated thermal conductivities for our model FePt structures as a function of temperature. (**b**) Local phonon density of states of the A1 disordered and L1_0_ ordered structures.

To understand the reduction in thermal conductivity from the L1_0_ phase to the A1 phase and gain further confidence in our experimental measurements, we perform MD simulations on model FePt systems defined by the different phases (details of the structure and potential used for the MD simulations are described in the methods section). Figure 4a shows the temperature dependent thermal conductivities for our model FePt systems. In comparison to the MD-simulated thermal conductivities of the cubic A1 phase, the thermal conductivities for the L1_0_ phase is greater at lower average temperatures and converges to the values predicted for the A1 phase as the average temperature of the simulations is increased. This observation is in good qualitative agreement with our experimental observation of a 23% increase in the thermal conductivity of the L1_0_ phase as compared to that of the A1 phase at RT and convergence of the measured thermal conductivities at higher temperatures between the two phases of FePt. This agreement suggests that the trends in thermal conductivities for the tetragonal L1_0_ phase and the cubic A1 phase can be generalized to other alloys and not restricted to FePt. Note, the temperature range simulated for the model alloy structures is near and above the Debye temperature of FePt alloys, therefore, a qualitative comparison using classical MD is valid in this temperature range.

In the context of the kinetic theory of gases, the lattice thermal conductivity is given as *κ* = 1/3*Cv*^2^*τ*, where *C* is the volumetric heat capacity, *v* is the phonon group velocity and *τ* is the total phonon scattering time. For disordered bulk alloys, impurity scattering dominates the phonon scattering processes (and *τ* is generally associated with negligible temperature dependence^15^). Whereas for ordered alloys, due to the imposed periodicity, impurity scattering has negligible contribution to the total phonon scattering rate. Therefore, in the ordered state (at higher temperatures), the primary phonon scattering mechanism that leads to the reduction in *κ* is the multiple phonon scattering processes associated with anharmonic phonon scattering. Considering only Umklapp scattering processes, the scattering time is inversely proportional to temperature (*τ* ~ 1/*T* at high temperatures) as predicted by Klemens^16^, and consequently at elevated temperatures, the rate of Umklapp scattering in the ordered L1_0_ phase eventually becomes similar to that of impurity scattering in the disordered phase. This would lead to a convergence of the MD-predicted thermal conductivities of the two phases above a certain temperature, and in some cases (at very high temperatures), the thermal conductivity of the ordered phase might eventually be less than that of the disordered phase^6^. In this regard, the temperature trends from our MD simulations (Fig. 4a) lends insight into these different phonon scattering mechanisms that dictate thermal conductivity in our model LJ-based FePt alloys. As shown in Fig. 4a, the thermal conductivity for the cubic A1 phase does not show a temperature dependence suggesting that impurity scattering dominates thermal conductivity at these temperatures and anharmonic scattering has negligible contribution to the total thermal conductivity. In contrast, the MD-predicted thermal conductivity for the L1_0_ phase shows an inverse relation to temperature that is characteristic of Umklapp dominated conductivity, as mentioned before.

We also calculate the local phonon density of states (DOS) (as explained in refs 17 and 18) for the L1_0_ and A1 structures (as shown in Fig. 4b). Where the DOS for the A1 phase is relatively flat for most frequencies, the DOS for the L1_0_ ordered phase shows sharp peaks at ~2, 3 and 5 THz frequencies. A deep trough is also observed for the L1_0_ phase at ~4 THz due to the fact that a diatomic basis in an ordered system leads to phonon band gaps (driven by the presence of optical phonons), which are not present in the A1 structure due to the presence of disorder. The difference in the DOS between these structures (in conjunction with the different scattering mechanisms dominant in the two phases) further explains the observed variance in the temperature trends in thermal conductivity. It is interesting to note that even though the ordered and disordered alloys posses very different densities of states in their phononic spectrums, the thermal conductivities at higher temperatures converge, which is attributed to the varying contributions from impurity and Umklapp scattering processes as mentioned in the previous paragraph.

It is important to note that the MD simulations are strictly classical in nature and are generally valid near and above a material’s Debye temperature (where all the phonon modes available in the crystal are excited)^19^. In this regard, it is reasonable to qualitatively compare the vibrational properties of the FePt alloys to our LJ-based models for the temperature range simulated in this work as these temperatures are near and above the Debye temperatures of FePt alloys^20^. Moreover, the parameters used in the LJ potential correctly reproduce the bandwidth of frequencies available in FePt alloys (<9 THz)^21^. It is also important to note that the MD simulations only consider the vibrational transport, and therefore, our experimentally measured temperature trends that demonstrate increasing *κ* with temperature for the L1_0_ and A1 phases can not be directly compared to the simulations due to the contribution of electronic transport to the experimentally measured *κ* for these FePt films. However, we can gain further qualitative insight into the relative contributions of electronic relaxation rates to thermal transport in the two phases by examining the TDTR data in the early pump-probe time regime (immediately after short pulsed absorption in these films). This will also allow us to assess the validity of our hypothesis that the phonon thermal conductivities of ordered and disordered FePt alloys converge at high temperatures, in accordance with MD simulations.

## Electronic Relaxation

**Figure 5:**
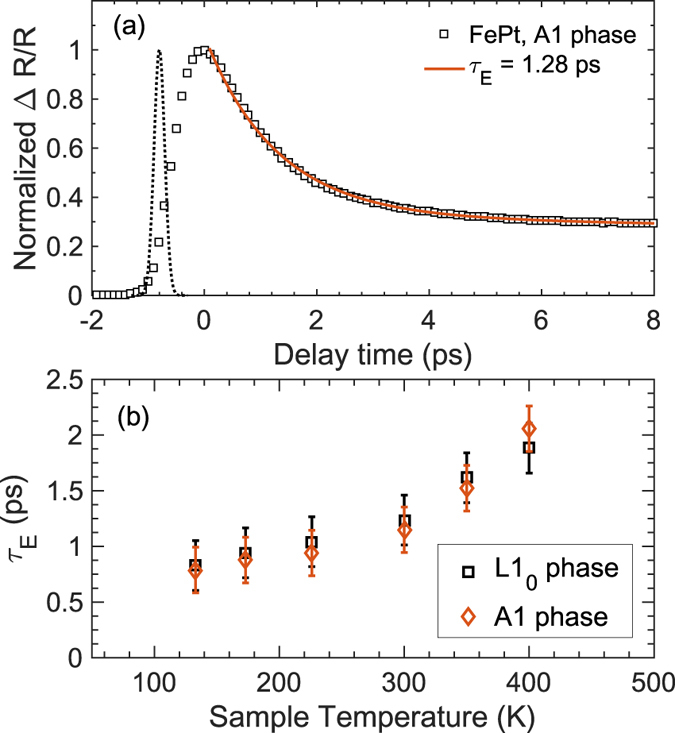
(**a**) Normalized magnitude of the in-phase and out-of-phase signals for the A1 phase FePt thin film sample for the first 10 ps during and after pulse absorption along with the biexponential fit to the experimental data. The cross-correlation between the pump and probe pulses is also shown (dotted curve). (**b**) Relaxation time constants extracted for the tetragonal L1_0_ phase and the cubic A1 phase at various sample temperatures.

To understand the varying electronic scattering mechanisms in the out-of-plane direction of the disordered and ordered phases, we consider the first few picoseconds of the thermoreflectance response after laser pulse absorption directly onto the FePt surfaces. Typically after short-pulsed excitation of a thin metal film on a dielectric substrate, the electron gas in a metal can be heated to several thousand degrees above the lattice temperature due to the large differences in the carriers’ heat capacities^22^,^23^,^24^. Therefore, the use of the conventional Fourier theory is no longer applicable at these short-time scales as electrons and phonons are out of thermal equilibrium and have to be considered as two separate channels of energy transport^24^,^25^. We study this short-time regime due to the fact that the electronic relaxation mechanisms in metals and metallic alloys occur within a couple of picoseconds, and are driven by various electronic scattering mechanisms (such as electron-magnon, electron-defect and electron-phonon coupling) that influence the electronic mobility^26^,^27^. Furthermore, the fact that these films are thicker than the optical penetration depth implies that electronic diffusion (also related to the electrical resistivity) away from the film surface could potentially affect the electronic relaxation times. Therefore, these measurements provide a semi-quantitative metric of the overall electronic relaxation time associated with the ordered and the disordered films in this section as discussed in the following paragraphs. We also note that, unlike our experimental procedure to measure the overall thermal conductivity of the samples, our pump and probe beams are incident on the surface of the FePt samples without the Al transducer layer. A characteristic thermoreflectance signal for the A1 phase of the FePt sample during and after laser pulse absorption is shown in Fig. 5a.

In general, for metallic systems, the electron-phonon relaxation mechanisms are studied under the framework of the two-temperature model (TTM). However, this model requires the knowledge of various thermophysical properties of the systems such as the temperature dependent heat capacity, electron-phonon coupling and thermal conductivities of the carriers^22^,^24^,^28^. Due to the fact that the electronic heat capacities and the electron-phonon coupling factors for our FePt samples are unknown, we do not attempt to compare the TTM to our experimental data and instead we consider the relative changes in the measured relaxation rates between the A1 and the L1_0_ phases as mentioned above. To this end, we fit a bi-exponential decay function to the magnitude of the in-phase and out-of-phase signals (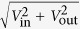) at early pump-probe time delayes (as shown in Fig. 5a) to extract the electron relaxation rate, *τ*_E_, which represents the fast relaxation due to electronic scattering events. The longer relaxation rate is associated with phonon-phonon driven relaxation mechanisms and does not influence the determination of the fast transient decay that is typically associated with electronic relaxation in metals. Figure 5b shows the relaxation rates obtained by this procedure for the A1 and the L1_0_ phases at various sample temperatures. As is clear, the electron relaxation rates for the two samples are nearly identical at all temperatures, suggesting that the electronic scattering mechanisms in the tetragonal as well as the cubic structures are similar at these time regimes. The relaxation rate of ~1 ps at RT is in agreement with that for 3 nm-thick continuous FePt thin layer reported in ref. 29.

Even though the electronic scattering rates are similar for the two phases, the measured in-plane electrical resistivities are highly dissimilar between the phases^30^. We note that we do not attempt to quantitatively separate the specific contributions from electrons and phonons to the total thermal conductivity of these phases due to the fact that measuring the electronic resistivity (and therefore the electronic contribution to the thermal conductivity) in the out-of-plane direction of these films is beyond the scope of this study. Even though the A1 phase has cubic symmetry and the in-plane electronic resistivity values can be assumed for the out-of-plane direction, a similar assumption can not be made for the L1_0_ structure due to the high anisotropy of this phase.

## Conclusions

In summary, we have measured the out-of-plane thermal conductivities and electronic relaxation rates for tetragonal L1_0_ FePt (001) easy-axis and cubic A1 FePt thin films via time-domain thermoreflectance over wide temperature range (133–500 K). The thermal conductivity of the ordered L1_0_ film is greater than that of the disordered A1 film below room temperature and the thermal conductivities of the two phases converge at higher temperatures. Electronic relaxation rates in the two phases are similar even though the in-plane electrical conductivity measurements are different. MD simulations of model Lennard-Jones FePt structures allude to the fact that the difference at relatively low temperatures and the eventual convergence at higher temperatures in the thermal conductivities between the two phases of FePt can be generalized to other binary alloys.

## Methods

### Time Domain Thermoreflectance

For our TDTR measurements, the samples are metalized with a 55 nm Al transducer layer. In our two-tint TDTR setup, sub-picosecond laser pulses emanate from a Spectra Physics Tsunami oscillator with a repetition rate of 80 MHz. The pulses are separated into a pump beam (that heats the sample) and a probe beam (that reflects off of the sample and allows for the accurate measurement of the change in thermoreflectance of the sample due to the heating event induced by the pump pulses). The pump beam is further modulated at 8.4 MHz through an electro-optic modulator and we monitor the in-phase (*V*_in_) and out-of-phase (*V*_out_) signals via a lock-in amplifier as a function of the probe delay time. The 1/*e*^2^ radii of our pump and probe spots at the sample surface are 30 and 10 *μ*m at the sample surface, respectively. The corresponding FWHM (full width at half maximum) of the pump and probe pulses measured via frequency-resolved optical gating technique are 164 fs and 152 fs, respectively^31^. This ensures the thermoreflectance signal is minimally affected by in-plane heat diffusion and the majority of the change in the signal is due to out-of-plane thermal transport. Other pertinent details of our specific experimental setup can be found in ref. 32 and the thermal model used to analyze the TDTR data is explained in refs 12, 13, 14.

### Molecular Dynamics Simulations

**Figure 6:**
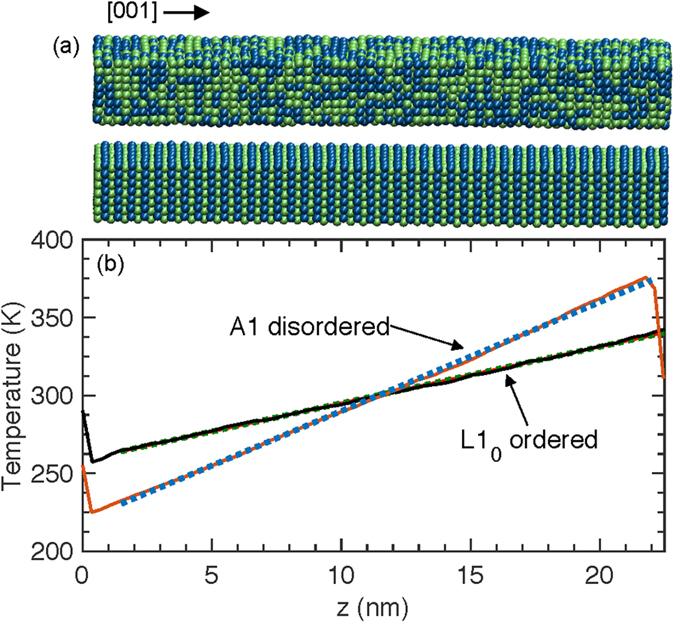
(**a**) Schematic of the A1 disordered (Top panel) and L1_0_ ordered model FePt computational domains. (**b**) Temperature gradient due to a steady-heat flux along the (001) direction.

For the tetragonal L1_0_ configuration, a Fe atom is placed at (0, 0, 0) and a Pt atom at 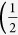, 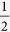, 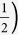 with regard to the conventional lattice vectors of the tetragonal unit cell. This configuration leads to a layered 1 × 1 superlattice structure in the c-axis direction as shown in the top panel of Fig 6a. The interaction between the atoms is defined by the 6–12 Lennard Jones (LJ) potential given by, 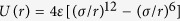, where *U* is the interatomic potential, *r* is the interatomic separation, and *σ* and *ε* are the LJ length and energy parameters, respectively. The values for the energy and length parameters for the interaction between and within the Fe and Pt atoms are taken from ref. 33. These parameters reproduced the structure of Fe, Pt and FePt and their melting points by MD simulations^33^. However, we note that the purpose of our simulations is to gain more insight on the relative effects of A1 and L1_0_ phases on thermal transport as opposed to specific properties of materials. Therefore, the LJ interatomic potential will be sufficient to provide the necessary qualitative understanding of the vibrational properties of the FePt alloys.

The cross sectional area of the simulation cell is 27 × 27 Å^2^ and the length in the z-direction is ~220 Å. The cutoff distance for the LJ potential is set to 9 Å for computational efficiency. The computational domains for our model LJ FePt alloys with the cubic (top panel) and tetragonal (bottom panel) phases are shown in Fig. 6a. The structures are relaxed under the Nose-Hoover thermostat and barostat (at zero pressure and the desired simulation temperature) for a total of 2 × 10^6^ time steps with a time step of 0.5 fs. After relaxation, we perform non-equilibrium MD simulations by applying a steady state heat flux that induces a temperature gradient along the z-direction as shown in Fig. 6b. For this purpose, we remove the thermostat and barostat and perform NVE integration (number of particles, volume and energy held constant – microcanonical ensemble) while adding fixed amount of energy per time step to a warm bath at one end and removing equal amount of energy from a cool bath at the other end. The thermal conductivities of the A1 and L1_0_ phases are calculated by invoking the Fourier law, *Q* = −*κ*∂*T*/∂*z*, where *Q* is the applied flux. We note that changing the heat flux did not alter the MD-simulated thermal conductivities.

## Additional Information

**How to cite this article**: Giri, A. *et al*. Influence of chemical ordering on the thermal conductivity and electronic relaxation in FePt thin films in heat assisted magnetic recording applications. *Sci. Rep*. **6**, 32077; doi: 10.1038/srep32077 (2016).
